# Prognosis analysis of patients with pancreatic neuroendocrine tumors after surgical resection and the application of enucleation

**DOI:** 10.1186/s12957-020-02115-z

**Published:** 2021-01-12

**Authors:** Junzhang Chen, Yongyu Yang, Yuanhua Liu, Heping Kan

**Affiliations:** Department of Hepatobiliary Surgery, Nanfang Hospital, Southern Medical University, 1838 Guangzhou North Ave, Guangzhou, 510515 Guangdong Province China

**Keywords:** Pancreatic neuroendocrine tumor (pNETs), Surveillance, epidemiology, end results (SEER) database, Surgical resection, Prognostic factor, Enucleation

## Abstract

**Objective:**

To investigate the prognostic factors of patients with pancreatic neuroendocrine tumor (pNETs) after surgical resection, and to analyze the value of enucleation for pNETs without distant metastasis that are well-differentiated (G1) and have a diameter ≤ 4 cm.

**Methods:**

Data from pNET patients undergoing surgical resection between 2004 and 2017 were collected from the Surveillance, Epidemiology, and End Results (SEER) database. Kaplan–Meier analysis and log-rank testing were used for the survival comparisons. Adjusted HRs with 95% CIs were calculated using univariate and multivariate Cox regression models to estimate the prognostic factors. *P* < 0.05 was regarded as statistically significant.

**Results:**

This study found that female, cases diagnosed after 2010, and pancreatic body/tail tumors were protective factors for good survival, while histological grade G3, a larger tumor size, distant metastasis, AJCC 8th stage III-IV and age over 60 were independent prognostic factors for a worse OS/CSS. For the pNETs that were well-differentiated (G1) and had a tumor diameter ≤ 4 cm, the type of surgery was an independent factor for the long-term prognosis of this group. Compared with pancreaticoduodenectomy and total pancreatectomy, patients who were accepted enucleation had better OS/CSS.

**Conclusion:**

For pNETs patients undergoing surgical resection, sex, year of diagnosis, tumor location, pathological grade, tumor size, distant metastasis, race, and age were independent prognostic factors associated with the OS/CSS of patients. For pNETs patients with G1 and a tumor diameter less than 4 cm, if the tumor was located over 3 mm from the pancreatic duct, enucleation may be a wise choice.

## Introduction

Neuroendocrine tumors (NETs) are rare tumors that generally originate from neuroendocrine cells in various organs, and the pancreas is one of the most common sites of NETs [1, 2]. Pancreatic neuroendocrine tumors (pNETs) constitute approximately about 3-5% of the total number of pancreatic tumors, and these tumors exhibit high heterogeneity. Compared with pancreatic adenocarcinomas, pNETs are relatively slow growing but have the potential to be malignant and develop distant metastases, most commonly in the liver [3]. In recent years, the incidence of pNETs in the population has shown a continuous upward tendency. Based on whether the tumors can secrete hormones to trigger clinical symptoms, they are divided into two types of tumors, functioning or nonfunctioning tumors. Functioning pNETs generally produce one or more biologically active peptides, such as insulin, somatostatin, gastrin, or glucagon, inducing specific clinical symptoms. They account for 30% of all pNETs [4]. Nonfunctioning tumors remain asymptomatic in the early stages and are often only detected at a late stage. Many findings have suggested that patients with non-functioning tumors have a worse prognosis [2, 5, 6]. At present, many studies have researched the prognostic factors of pNETs all around the world. However, there are still many limitations and controversies about the prognostic factors of this disease [6–8]. There are few large databases exclusively researching the prognostic factors of pNET patients nonradical surgical resection. Surgery is the only treatment that can cure the disease. While pancreatic surgery is quite difficult, and there is a high risk of complications during and after surgery, pancreatic neuroendocrine tumors exhibit great heterogeneity [2]. These differences and lack of prognostic factors make the choice of surgical procedure for pNETs more controversial. pNETs’ operations can be divided into two categories: one is tissue-sparing resection, such as enucleation and middle segmental pancreatic resection; the other is regular pancreatectomy (standard pancreatic resection), generally including pancreaticoduodenectomy, partial pancreatectomy, and total pancreatectomy. A few studies have shown that compared to the other operations, the procedure of enucleation is simpler, has a shorter operation time, fewer postoperative complications, and the patients recover faster after surgery [9–11]. It is now generally accepted that the indications for enucleation are diameter of the tumor < 2 cm and the tumor is at least 3 mm away from the main pancreatic duct. However, there is limited research to support surgical decisions. This paper retrospectively analyzed the 2571 pNETs cases of radical or non-radical surgical resection that were collected from 2004 to 2017, using SEER data, to study the prognostic factors of these patients undergoing surgical resection. Another important aspect of the study was to explore the long-term efficacy of enucleation in pNET patients with pathological grade G1 and a tumor diameter ≤ 4 cm.

## Materials and methods

### Data source

The case data were derived from the SEER database of the National Cancer Institute of the USA, using the SEER stat software (version 8.3.6), and the reference number was 11706-Nov2019. This database covers approximately 30.0% of cancer cases in the US population and provides clinical workers with first-hand data about cancer epidemiology, clinical features, treatment information, and outcomes [3]. All of the pNETs cases were diagnosed from 2004 to 2017. The follow-up cutoff date was December 31, 2017. The range of follow-up time was 1 ~ 167 months. This study was approved by the Institutional Review Board.

### Inclusion and exclusion criteria

The inclusion criteria were (1) pancreatic neuroendocrine tumor was the only primary tumor. (2) Pancreatic anatomical sites were included (C25.0–C25.8) in our study. (3) ICD-O-3 (International Classification of Diseases for Oncology, 3rd edition) morphology codes 8150-8153, 8155-8157, 8240-8243, and 8246, 8249 were selected, and then 5240 pNET patients were collected from the SEER database.

**Fig. 1:**
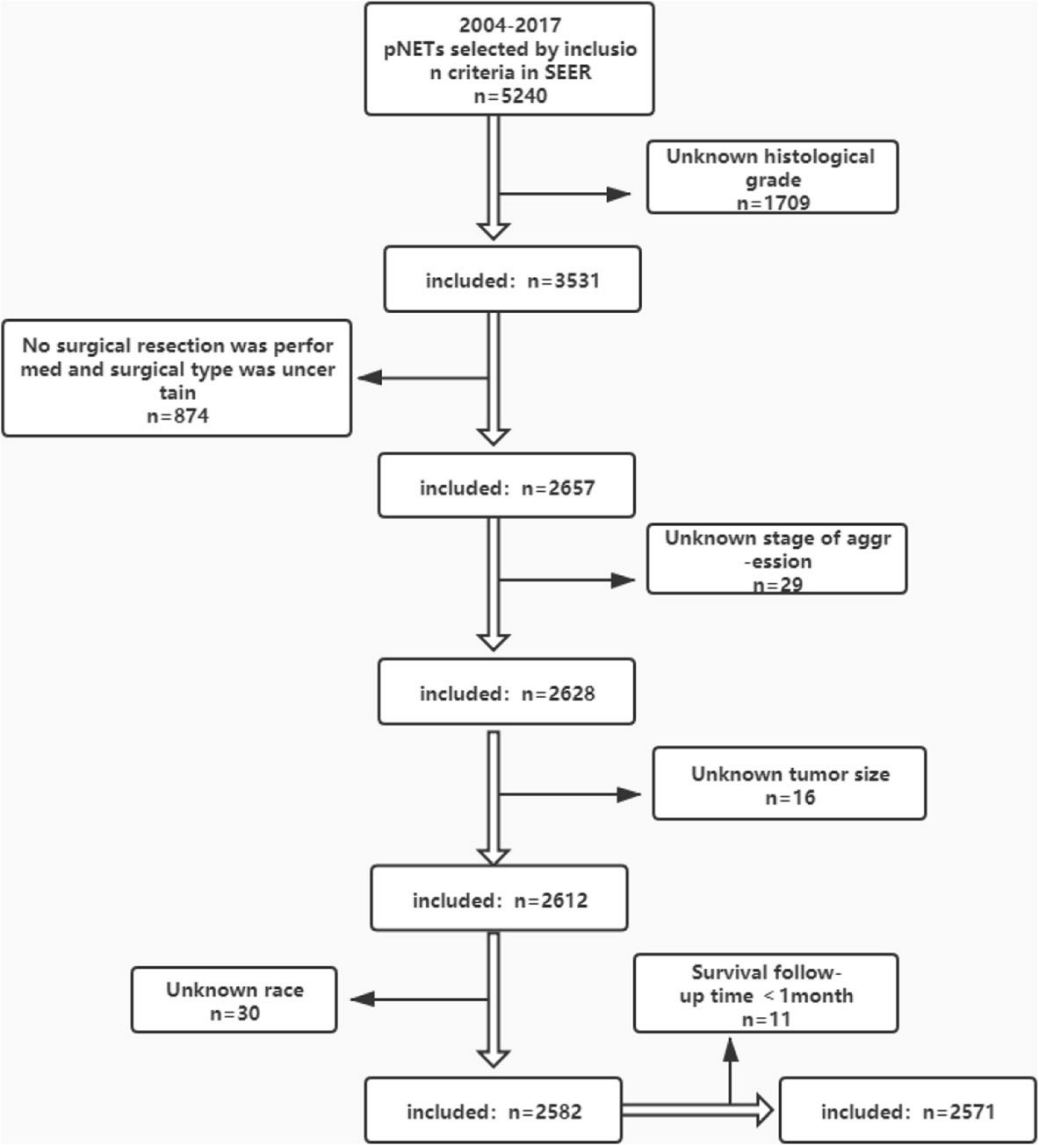
Exclusion criteria and study design

The exclusion criteria are described below, and shown in Fig. 1.

1. Cases with missing data (*n* = 2669)

I. Unknown histological grade (*n* = 1709)
II. No surgical resection was performed and surgical type was uncertain (*n* = 874)
III. Unknown stage of aggression (*n* = 29)
IV. Unknown tumor size (*n* = 16)
V. Unknown race (*n* = 30)

2) Cases with survival time < 1 month (*n* = 11)

After exclusion, a total of 2571 patients were included. Variables included in the study had sex, year of diagnosis, age, race, tumor size, histological grade, AJCC 8th stage, type of surgery, distant metastasis, lymph node metastasis, type of tumor, and location of the tumor. There are a few data limitations in the SEER database, such as which histological methods were used for grading. In this study, well-differentiated was classified as G1; moderated-differentiated was classified as G2; poorly differentiated and undifferentiated were classified as G3. Based on the tumor size, lymph nodes, and distant metastasis, the 2571 patients were restaged using the AJCC 8th stage standards. Overall survival (OS) was censored according to “vital status record.” Cancer-specific survival (CSS) was based on “cancer-specific death.”

### Statistical analysis

Univariate and multivariate Cox proportional hazard analysis was used to identify the factors that were independently associated with CSS and OS. Variables with *P* < 0.1 on univariate analysis were further included in the multivariate analysis. The Kaplan–Meier analysis with the log-rank test was used to describe the CSS and OS. All tests were two-sided, *P* < 0.05 was considered as statistically significant. All statistical analyses were performed using IBM SPSS Statistics version 24.0, and survival curves were drawn with GraphPad Prism 8.0.1.

## Result

### Baseline characteristics of the patients

After screening against the exclusion criteria, 2571 patients were included in the study. The detailed clinical characteristics of the patients are shown in Table 1. Male patients accounted for 51.8% of the total. A marked increase in the number of pNETs cases after 2010 was seen, accounting for 85% of the total number of cases. Approximately 14.6% of the patients had metastasis before surgical resection, and 16.6% of the patients had no lymph node dissection during the operation. The number of white people accepting surgery was much higher than that of the other races.

**Table 1:** Baseline characteristics of p-NET patients with radical or non-radical resection

Characteristics	*n*	%
Gender		
Male	1332	51.8
Female	1239	48.2
Year of diagnosis		
2004-2009	379	14.7
2010-2017	2192	85.3
Location of tumor		
Head of pancreas	823	32.0
Body/tail of pancreas	1486	57.8
Other^a^	262	10.2
Type of tumor		
Functional	200	7.8
Non-functional	2371	92.2
Histological grade		
G1	1915	74.5
G2	511	19.9
G3	145	5.6
Distant metastasis		
No	2196	85.4
Yes	375	14.6
Tumor size		
≤ 2 cm	951	37.0
2-4 cm	920	35.8
> 4 cm	700	27.2
AJCC 8^th^ stage		
I-II	1098	42.7
III-IV	1103	42.9
Unknown	367	14.4
Lymph node metastasis		
Yes	740	28.8
No	1405	54.6
Untested^b^	426	16.6
Race		
White	1971	76.7
Black	316	12.3
Other^c^	284	11.0
Age at diagnosis (year)		
≤ 60	1583	61.6
> 60	988	38.4

^a^Overlapping lesion of the pancreas, pancreatic duct, and other specified parts of pancreas

^b^No lymph nodes were detected during surgery

^c^Asian/American Indian/Alaska Native/Pacific Islander

### Survival outcomes

Table 2 showed that the log-rank test was used to analyze the factors associated with CSS and OS in 2571 pNETs cases. It can be seen that except for the type of tumor, sex, year of diagnosis, tumor location, pathological grade, preoperative distant metastasis, tumor size, AJCC stage, lymph node metastasis, race, and age were associated with the OS. Race was a prognostic factor for OS but not CSS. The CSS of the patients were related to sex, year of diagnosis, tumor location, pathological grade, preoperative distant metastasis, tumor size, AJCC stage, lymph node metastasis, and age. The 5-year OS was 85.6% for all cases, the 5-year CSS was 88.6%, and the median follow-up time was 37 m. The median survival time was not available.

**Table 2:** The calculation of survival time using Kaplan-Meier curves and log-rank test

Characteristics	5 years OS (%)	*P*	Median follow-up, time (month)	5 years CSS (%)	*P*
Gender		0.001			0.020
Male	82.6		37	86.9	
Female	88.6		37	90.9	
Year of diagnosis		0.000			0.000
2004-2009	77.4		105	80.1	
2010-2017	88.0		32	91.2	
Location of tumor		0.000			0.001
Head of pancreas	82.9		38	86.3	
Body/tail of pancreas	88.3		36	91.0	
Other^a^	79.9		40	83.5	
Type of tumor		0.240			0.182
Functional	81.4		65	85.7	
Non-functional	86.1		35	89.0	
Histological grade		0.000			0.000
G1	89.2		39	92.3	
G2	84.3		36	87.3	
G3	46.1		23	47.6	
Distant metastasis		0.000			0.000
No	90.1		37	93.5	
Yes	64.1		40	65.5	
Tumor size		0.000			0.000
≤ 2 cm	93.6		34	96.7	
2-4 cm	83.2		37	86.4	
> 4 cm	79.5		42	82.2	
AJCC 8^th^ stage		0.000			0.000
I-II	93.7		35	96.9	
III-IV	76.4		41	79.0	
Unknown	95.1		33	96.5	
Lymph node metastasis		0.000			0.000
Yes	76.2		41	78.5	
NO	90.4		36	94.3	
Not examined^b^	88.8		33	90.4	
Race		0.029			0.069
White	85.0		38	88.0	
Black	87.1		33	90.1	
Other^c^	88.2		37	91.1	
Age at diagnosis (year)		0.000			0.004
≤ 60	88.0		40	90.1	
> 60	81.4		33	86.1	

^a^Overlapping lesion of the pancreas, pancreatic duct, and other specified parts of pancreas

^b^No lymph nodes were detected during surgery

^c^Asian/American Indian/Alaska Native/Pacific Islander

### The confirmation of independent prognosis factors

Tables 3 and 4 show the univariate and multivariable regression analysis results. The variables *P* < 0.1 in Cox univariate analysis were further analyzed using multivariate analysis. The results showed that female patients, diagnosed after 2010, and tumors of the pancreatic body and tail had better long-term survival. If the pathological grade was G3, there was distant metastasis, the diameter of the tumor was larger, AJCC 8th stage III-IV, the white race, and older age were associated with worse OS/CSS. Sex, year of diagnosis, location of the tumor, histological grade, distant metastasis, tumor size, AJCC 8th Stage, race, and age were the independent prognostic factors for pNET patients undergoing surgical resection.

**Table 3:** Univariate and multivariable Cox proportional hazards regression model for OS in 2571 p-NET patients

Characteristics	Univariate analysis		Multivariate analysis	
	Hazard ratio (95% CI)	*P*	Hazard ratio (95% CI)	*P*
Gender				
Male	1 (reference)		1 (reference)	
Female	0.690 (0.554-0.861)	0.001	0.692 (0.553-0.867)	0.001
Year of diagnosis				
2004-2009	1 (reference)		1 (reference)	
2010-2017	0.502 (0.393-0.642)	0.000	0.715 (0.558-0.916)	0.008
Location of tumor				
Head of pancreas	1 (reference)		1 (reference)	
Body/tail of pancreas	0.638 (0.506-0.806)	0.000	0.651 (0.510-0.830)	0.001
Other*	0.928 (0.65-1.307)	0.670	0.992 (0.696-1.413)	0.965
Type of tumor				
Functional	1 (reference)			
Non-functional	0.829 (0.606-1.134)	0.241		
Histological grade				
G1	1 (reference)		1 (reference)	
G2	1.504 (1.138-1.988)	0.004	1.145 (0.861-1.522)	0.352
G3	7.033 (5.392-9.173)	0.000	3.949 (2.977-5.240)	0.000
Distant metastasis				
No	1 (reference)		1 (reference)	
Yes	3.992 (3.206-4.970)	0.000	2.116 (1.628-2.751)	0.000
Tumor size				
≤ 2 cm	1 (reference)		1 (reference)	
2-4 cm	2.451 (1.759-3.416)	0.000	1.452 (1.018-2.071)	0.039
> 4 cm	3.500 (2.526-4.850)	0.000	1.517 (1.055-2.183)	0.025
AJCC 8^th^ stage				
I-II	1 (reference)		1 (reference)	
III-IV	3.780 (2.834-5.043)	0.000	1.644 (1.091-2.476)	0.017
Unknown	1.058 (0.635-1.760)	0.829	0.671 (0.333-1.351)	0.264
Lymph node				
Metastasis	1 (reference)		1 (reference)	
Yes	0.387 (0.304-0.492)	0.000	0.908 (0.664-1.241)	0.544
No	0.540 (0.390-0.747)	0.000	1.635 (1.067-2.507)	0.024
Not examined†				
Race				
White	1 (reference)		1 (reference)	
Black	0.959 (0.686-1.342)	0.809	1.160 (0.825-1.630)	0.394
Other‡	0.545 (0.346-0.859)	0.009	0.583 (0.368-0.924)	0.022
Age at diagnosis (year)				
≤ 60	1 (reference)		1 (reference)	
> 60	1.583 (1.272-1.969)	0.000	1.550 (1.239-1.938)	0.000

Univariate analysis of variables with *P* < 0.1, then further analysis by multivariate cox regression model

*Overlapping lesion of the pancreas, pancreatic duct, and other specified parts of pancreas

†No lymph nodes were detected during surgery

‡Asian/American Indian/Alaska Native/Pacific Islander

**Table 4:** Univariate and Multivariable cox proportional hazards regression model for CSS in 2571 p-NET patients

Characteristics	Univariate analysis		Multivariate analysis	
	Hazard ratio (95% CI)	*P*	Hazard ratio (95% CI)	*P*
Gender				
Male	1 (reference)		1 (reference)	
Female	0.746 (0.582-0.955)	0.020	0.745 (0.578-0.960)	0.023
Year of diagnosis				
200-2009	1 (reference)		1 (reference)	
2010-2017	0.411 (0.313-0.540)		0.640 (0.487-0.842)	0.001
Location of tumor				
Head of pancreas	1 (reference)		1 (reference)	
Body/tail of pancreas	0.609 (0.468-0.792)	0.000	0.624 (0.472-0.823)	0.001
Other^a^	0.948 (0.648-1.388)	0.785	1.026 (0.690-1.525)	0.900
Type of tumor				
Functional	1 (reference)			
Non-functional	0.789 (0.556-1.119)	0.183		
Histological grade				
G1	1 (reference)		1 (reference)	
G2	1.729 (1.264-2.367)	0.000	1.230 (0.893-1.693)	0.204
G3	8.962 (6.707-11.975)	0.000	4.540 (3.337-6.177)	0.000
Distant metastasis				
No	1 (reference)		1 (reference)	
Yes	5.504 (4.308-7.032)	0.000	2.536 (1.899-3.387)	0.000
Tumor size				
≤ 2 cm	1 (reference)		1 (reference)	
2-4 cm	3.199 (2.116-4.836)	0.000	1.646 (1.060-2.555)	0.026
> 4 cm	4.895 (3.265-7.337)	0.000	1.711 (1.096-2.672)	0.018
AJCC 8^th^ stage				
I-II	1 (reference)		1 (reference)	
III-IV	6.158 (4.200-9.029)	0.000	1.956 (1.167-3.278)	0.011
Unknown	1.307 (0.682-2.505)	0.420	0.685 (0.298-1.572)	0.372
Lymph node				
Metastasis	1 (reference)		1 (reference)	
Yes	0.275 (0.206-0.366)	0.000	0.798 (0.551-1.131)	0.197
No	0.508 (0.356-0.725)	0.000	1.769 (1.138-2.750)	0.011
Not examined^b^				
Race				
White	1 (reference)		1 (reference)	
Black	0.938 (0.640-1.375)	0.742	1.132 (0.767-1.670)	0.533
Other^c^	0.554 (0.333-0.922)	0.023	0.573 (0.342-0.962)	0.035
Age at diagnosis (year)				
≤ 60	1 (reference)		1 (reference)	
> 60	1.439 (1.123-1.844)	0.004	1.395 (1.081-1.801)	0.011

Univariate analysis of variables with *P* < 0.1, then further analysis by multivariate Cox regression model

^a^Overlapping lesion of the pancreas, pancreatic duct, and other specified parts of pancreas

^b^No lymph nodes were detected during surgery

^c^Asian/American Indian/Alaska Native/Pacific Islander

### The evaluation of enucleation in pNETs

**Fig. 2:**
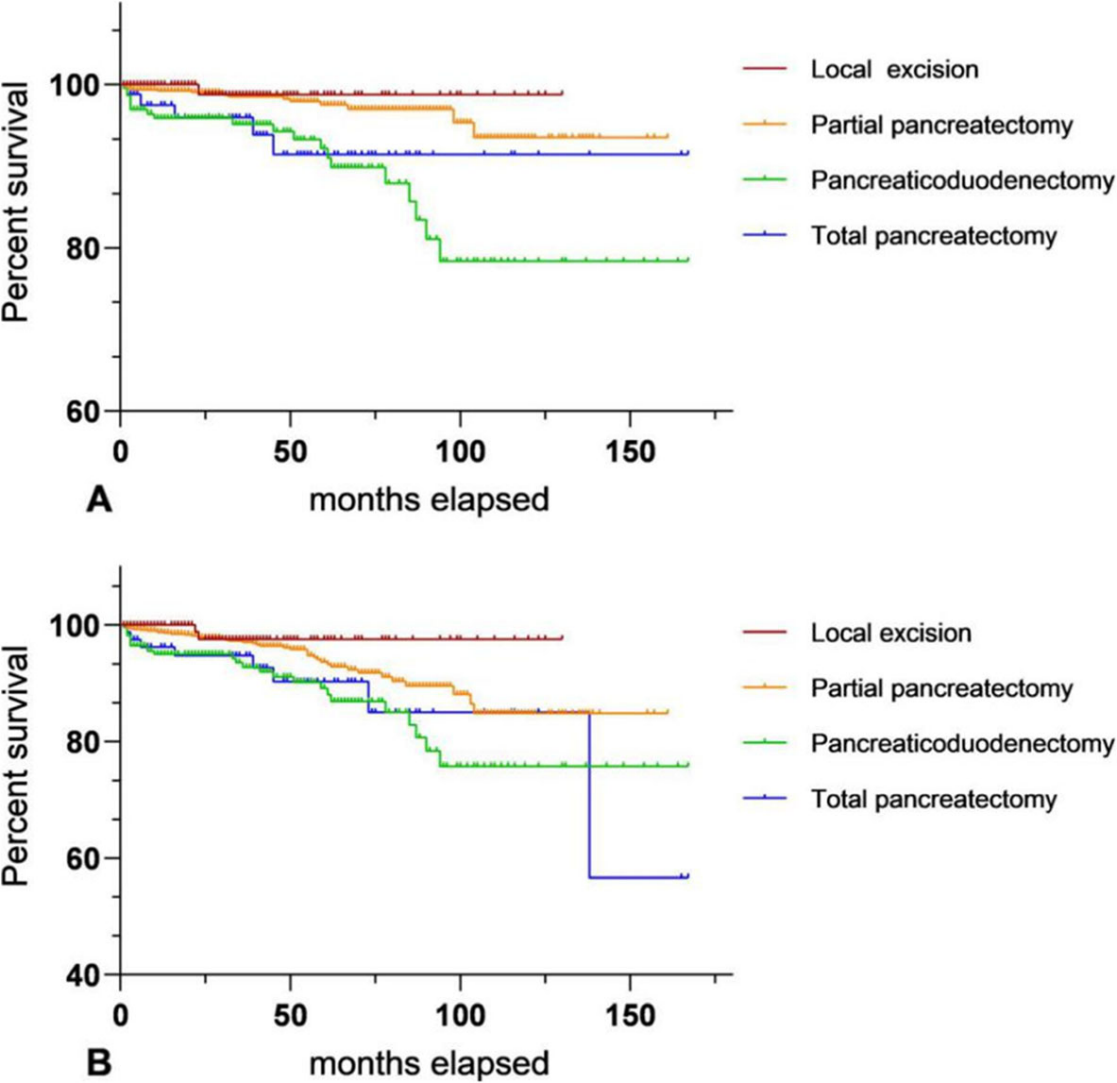
Comparison of long-term survival between different surgical procedures for G1 pNETs with diameter ≤ 4 cm; **a** (CSS of different operations); **b** (OS of different operations)

**Fig. 3:**
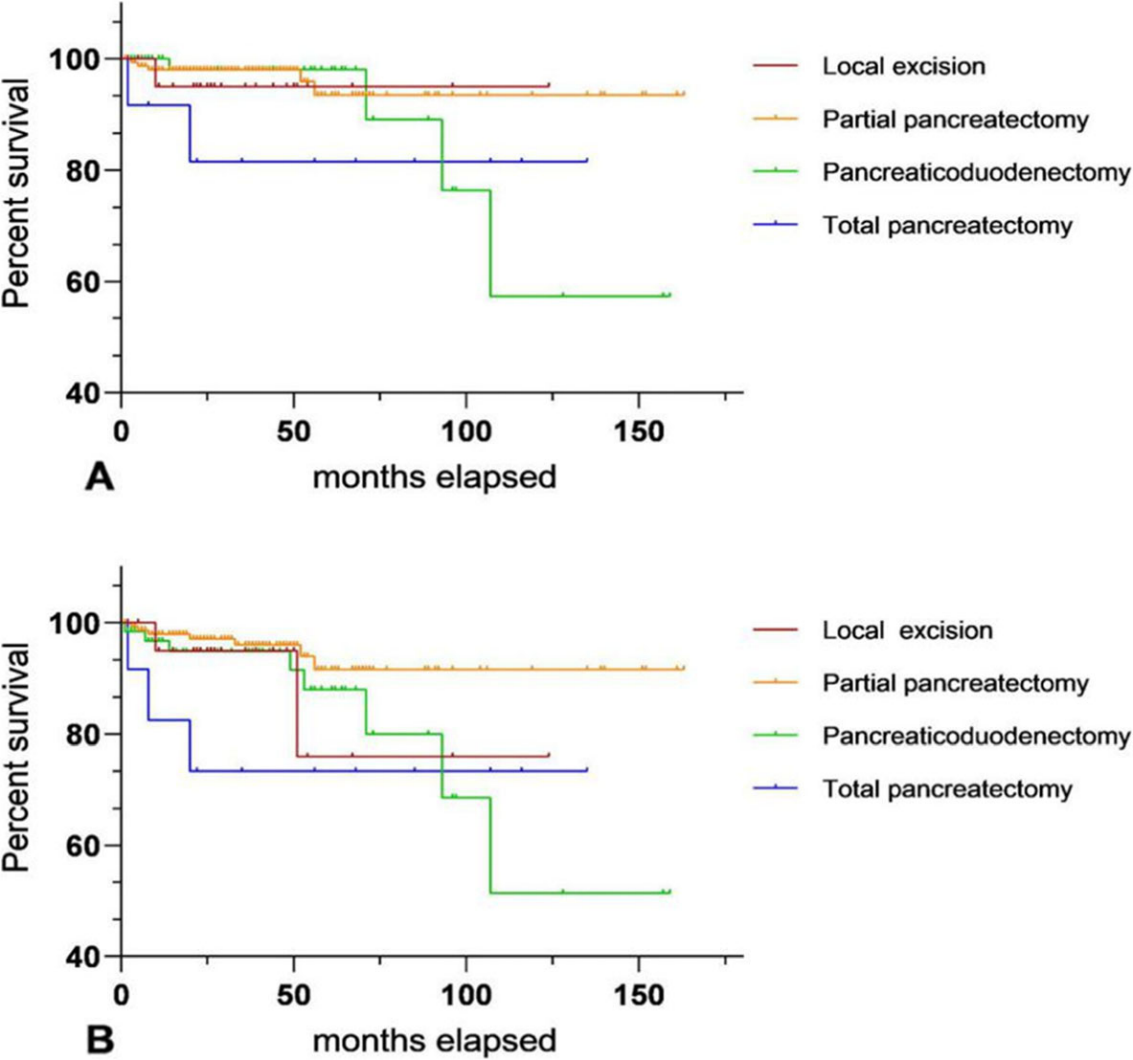
Comparison of long-term survival between different surgical procedures for G2 pNETs with diameter ≤ 4 cm; **a** (CSS of different operations); **b** (OS of different operations)

Figure 2 shows the survival curve among different surgical modalities for pNETs with ≤ 4 cm and G1, including OS and CSS, drawn by GraphPad Prism. Both of them *P* value < 0.05. Table 5 shows the multivariate Cox proportional hazard model analyzing long-term prognosis among different operations. The clinical variables brought into the analysis were independent prognosis factors for pNET patients who accepted surgical treatment, including sex, year of diagnosis, tumor location, AJCC 8th stage, race, and age. It can be seen that for pNETs with G1, diameter ≤ 4 cm, the mode of surgery is an independent factor for their long-term prognosis. Compared with enucleation, patients who received total pancreatectomy or pancreaticoduodenectomy had worse OS and CSS. The long-term survival of 273 pNETs with G2 and diameter ≤ 4 cm was analyzed by univariate regression analyses. The *P* value was over 0.1, so it could not be further analyzed by a multivariate Cox proportional hazard model. The OS and CSS of patients with partial pancreatic resection seemed to be better than for other procedures (Fig. 3).

**Table 5:** The Multivariable Cox proportional hazards regression model was applied to investigate the roles of different surgery on OS/CSS for G1 p-NETs with diameter ≤ 4 cm

Characteristics		Multivariate analysis (OS)	*P*	Multivariate analysis (CSS)	*P*
	*N* = 1387	Hazard ratio (95% CI)		Hazard ratio (95% CI)	
Gender					
Male	685	1 (reference)		1 (reference)	
Female	702	0.635 (0.403-1.001)	0.050	0.844 (0.470-1.516)	0.570
Year of diagnosis					
2004-2009	126	1 (reference)		1 (reference)	
2010-2017	1261	1.697 (0.868-3.316)	0.122	1.376 (0.591-3.203)	0.460
Location Of tumor					
Head of pancreas	406	1 (reference)		1 (reference)	
Body/tail of pancreas	854	0479 (0.260-0.881)	0.018	0.502 (0.213-1.187)	0.117
Other^a^	127	0.880 (0.397-1.952)	0.753	1.082 (0.376-3.111)	0.884
Surgery					
Enucleation	129	1 (reference)		1 (reference)	
Partial pancreatectomy	938	3.254 (0.747-14.176)	0.116	3.246 (0.403-26.158)	0.269
Pancreaticoduodenectomy	235	4.945 (1.080-22.637)	0.039	10.641 (1.270-89.181)	0.029
Total pancreatectomy	85	5.862 (1.210-28.396)	0.028	10.310 (1.164-91.352)	0.036
AJCC 8^th^ stage					
I	505	1 (reference)		1 (reference)	
II	282	1.989 (1.041-3.798)	0.037	1.766 (0.710-4.391)	0.221
III	310	1.774(0.951-3.312)	0.072	1.955 (0.858-4.455)	0.111
Unknown	290	1.742 (0.809-3.754)	0.156	2.385 (0.842-6.752)	0.102
Race					
White	1051	1 (reference)		1 (reference)	
Black	173	1.402 (0.731-2.689)	0.310	1.056 (0.410-2.720)	0.911
Other^b^	163	0.727 (0.330-1.601)	0.428	0.799 (0.310-2.057)	0.642
Age at diagnosis (year)					
≤ 60	844	1 (reference)		1 (reference)	
> 60	543	1.866 (1.192-2.921)	0.006	1.998 (1.107-3.803)	0.022

^a^Overlapping lesion of the pancreas, pancreatic duct, and other specified parts of the pancreas

^b^Asian/American Indian/Alaska Native/Pacific Islander

## Discussion

The pNET patients data were collected from the SEER database of the National Cancer Institute of the USA. After screening against the inclusion and exclusion criteria, 2571 pNET patients were selected. Different from other studies that analyzed the prognostic factors of pNET patients, this study was specific to pNET patients undergoing surgical resection to explore the relevant factors affecting the prognosis of these patients, increasing the clinical evidence for this rare disease. Another important aspect of this study was to clarify the application value of enucleation for pNETs which might promote the development of pNETs surgical treatment.

First, as we can see from Table 2, the results of log-rank analysis showed that, except for the type of tumor, sex, year of diagnosis, tumor location, pathological grade, distant metastasis, tumor size, AJCC 8th stage, lymph node metastasis, and patient’s age were all significantly associated with OS and CSS. Ethnicity was associated with patient OS but not CSS. In Tables 3 and 4, Cox multivariate analysis showed that women, diagnosed after 2010, and pancreatic body/tail tumor were independent protective factors for the long-term survival of pNET patients undergoing surgical resection, although the reason why women had a better OS/CSS was not clear.

Patients diagnosed after 2010 can receive better medical technical support. Surgical resection of the pancreatic body/tail is simpler than that of the pancreatic head. The former does not require the removal of a portion of the stomach and duodenum [12]. Patients may be more willing to undergo surgery without needing higher risk pancreaticoduodenectomy.

pNET patients with G3, distant metastasis, larger tumor diameter, AJCC 8th III-IV stage, the white race, or age > 60 years old were associated with a worse OS/CSS. A higher pathological grade and lower differentiation of tumor cells indicate the tumor may be more malignant [13, 14], some pNET patients with distant metastasis are difficult to treat since the removal of the primary and metastatic tumors may be impossible, and most of them can only achieve R1 or R2 resection [8]. Their prognosis is poorer than patients with an R0 resection. Some studies have shown that tumor size is not associated with the long-term prognosis of patients [1, 15]. However, this study found that tumor diameter was an independent factor affecting the OS/CSS of patients. A larger tumor may increase the difficulty of the operation, which also indicates that the tumor has existed for a long time, which increases the risk of distant metastasis of tumor cells. AJCC 8th staging is the latest pNETs staging method and its practicability has been widely recognized, and later staging means patients may have a poor prognosis [16]. Elderly patients are generally in poor health and more likely to have chronic diseases or perioperative complications, and consequently, older pNET patients have a worse prognosis. Although some studies suggested that race was an independent prognostic factor in pNETs, the overall prognosis of blacks was worse than the white race, which may be related to economic and social status [15, 17]. Among patients who had received surgical resection, the long-term prognosis between blacks and whites were not significant difference in this study, and white people had worse OS/CSS than that of other race such as Asian American. Many studies suggested that preoperative lymph node metastasis was associated with the long-term prognosis in pNET patients [18, 19], while others have not found a significant connection [20, 21]. In this study, Cox multivariate analysis suggested that lymph node metastasis could not be used as an independent prognostic factor for postoperative patients. Lymph node ratio (LNR) may be more persuasive as a prognostic factor than simply assessing the presence or absence of lymph node metastasis [22], but the specific mechanism needs to be supported by more multiple-center and large clinical database studies in the future. Comparing functional with nonfunctional tumors, the present clinical consensus is that nonfunctional tumors have a worse prognosis than functional tumors due to lack of clinical symptoms in the early stage. NF-pNETs are generally found at a later stage. However, with the progress in examination technology and the popularization of routine physical examinations, the influence of different tumor types on survival prognosis remains to be discussed. This study showed that there was no significant difference between functional and nonfunctional tumors for OS/CSS. It might be that the SEER database classified many early stage F-PNETs tumors as benign lesions and did not include them in the database, which affected the final statistical results. Another reason could be only pNET patients undergoing surgical resection were included in this study, and there may be no significant difference in the long-term prognosis between postoperative F-PNET and NF-PNET patients.

Current surgical principles consider that enucleation is only suitable for tumors less than 2 cm in diameter and the distance between the tumor and main pancreatic duct needs to be over 3 mm [11]. However, this point has not been verified by multiple-center and large clinical databases, and the research using small sample data is also scarce. The application value of enucleation for pNETs still need to be determined. This study specifically analyzed the value of enucleation for well and moderately differentiated pNETs with a diameter ≤ 4 cm. As shown in Table 5, pNETs with G1 and diameter ≤ 4 cm who received pancreaticoduodenectomy or total pancreatectomy had a worse OS/CSS than those undergoing enucleation. This may be the result of the higher risk of these two procedures and the higher probability of postoperative complications. On the other hand, well-differentiated pNETs have lower pNETs malignant potential [13], which can greatly increase the possibility of achieving a complete radical resection. There was no significant difference in OS/CSS between partial pancreatic resection and enucleation. However, some studies have confirmed that the procedure of enucleation is a relatively simple, short operation, with less trauma to the patients, and a shorter postoperative hospitalization time [10, 23]. Therefore, in general, for well-differentiated, and diameter ≤ 4 cm pNET patients without distant metastasis, enucleation seems to be more sensible in cases of radical resection. Figure 3 shows long-term survival curves of G2 pNET patients with a tumor diameter ≤ 4 cm. There was no statistical significance between groups in univariate Cox regression analysis. The primary cause of this may be the insufficient number of cases. Compared to G1 cases, the total number of G2 pNET patients with a diameter ≤ 4 cm included in the study was 273, and among them, only 23 patients were enucleated. Total pancreatectomy was performed in only 13 cases. The trend of the K-M survival curves suggests that enucleation has no significant advantage in long-term outcomes among G2 tumors and that patients receiving partial pancreatectomy seem to have better OS and CSS. However, more relevant studies need to prove this.

This present study has certain limitations. Firstly, it was retrospective study which could exist selection bias. Secondly, as a population-based registry, the SEER database has not provided some detailed information, such as the recurrence of p-NETs after surgery resection, postoperative complications, surgical margin status, and additional adjuvant therapy (chemotherapy, targeted, or endocrine therapy). Furthermore, the SEER database could not absolutely avoid coding errors due to extensive collection of case data from different regions. Despite the existence of above limitations, our research still produced important clinical values.

## Conclusion

This study has demonstrated the independent prognostic factors of pNET patients after surgical resection, including sex, year of diagnosis, location of the tumor, pathological grade, tumor size, distant metastasis, race, and age. The study found that for G1 pNET patients with a tumor diameter ≤ 4 cm, enucleation seems to be a better surgical protocol if the distance between the tumor and the main pancreatic duct is over 3 mm.

## Data Availability

The datasets analyzed in this study are collected from SEER repository (https://seer.cancer.gov/).
